# Genome-Wide Identification and Expression Analysis of the *NAC* Gene Family in Alfalfa Revealed Its Potential Roles in Response to Multiple Abiotic Stresses

**DOI:** 10.3390/ijms231710015

**Published:** 2022-09-02

**Authors:** Fei He, Lixia Zhang, Guoqing Zhao, Junmei Kang, Ruicai Long, Mingna Li, Qingchuan Yang, Lin Chen

**Affiliations:** 1 Institute of Animal Science, Chinese Academy of Agricultural Sciences, Beijing 100193, China; 2 Institute of Forage Crop Science, Ordos Academy of Agricultural and Animal Husbandry Sciences, Ordos 017000, China

**Keywords:** alfalfa, *MsNAC*, gene family, genome-wide, abiotic stress

## Abstract

NAC (NAM, ATAF1/2, and CUC2) transcription factors compose one of the largest families of plant-specific transcription factors; they are widely involved in plant growth and development and have especially important roles in improving stress resistance in plants. However, *NAC* gene family members in alfalfa (*Medicago sativa* L.) have not been systematically identified and analyzed genome-wide due to the complexity of the alfalfa reference genome. In this study, a total of 421 *M. sativa* *NAC* genes (*MsNAC*s) were identified from the alfalfa “Xinjiangdaye” reference genome. Basic bioinformatics analysis, including characterization of sequence length, protein molecular weight and genome position and conserved motif analysis, was conducted. Expression analysis showed that 47 *MsNACs* had tissue-specific expression, and 64 *MsNACs* were expressed in all tissues. The transcriptomic profiles of the genes were very different, indicating that these *MsNACs* have various functions in alfalfa growth and development. We identified 25, 42 and 47 *MsNACs* that respond to cold, drought and salt stress based on transcriptome data analysis and real-time quantitative PCR (RT–qPCR). Furthermore, 22 *MsNACs* were found to respond to both salt and drought stress, and 15 *MsNACs* were found to respond to cold, salt and drought stress. The results of this study could provide valuable information for further functional analysis of *MsNACs* and for the improvement of stress resistance in alfalfa.

## 1. Introduction

Plants living in nature are exposed to biotic and abiotic stresses at all times [1]. In the process of adapting to their environment, plants regulate the coordinated expression of a large number of genes, including transcription factors [1]. Transcription factors can act by either activating or repressing the expression of other genes [2]. The NAC transcription factor family is one of the largest plant-specific transcription factor families [3,4]. The NAC transcription factors have a general structure that consists of a highly conserved N-terminal domain involved in DNA binding (called the NAC domain) and a C-terminal region highly divergent in sequence and length that functions as the activation domain [5,6]. The N-terminal domain is closely associated with nuclear localization and recognition and the binding of DNA sequences of downstream target genes, while the C-terminus has a transcriptional activation or transcriptional repression function [5,6].

NAC transcription factors regulate the entire process of plant growth and development, including the formation of the plant secondary wall and xylem, root growth, fruit ripening, and leaf senescence [7]. In *Arabidopsis thaliana*, the NAC transcription factor *SND1* (secondary wall-associated NAC domain protein 1) regulates secondary wall and xylem formation by activating the expression of secondary wall-related genes [8]. In strawberry, the NAC transcription factor *FaRIF* (ripening inducing factor) is a core transcription factor in the regulation of strawberry fruit ripening and is involved in several fruit ripening regulatory pathways, including phytohormone regulation pathways [9]. In *Citrullus lanatus*, *ClNAC68* positively regulates the accumulation of IAA during fruit ripening and promotes normal seed development by directly binding to the promoter region of the IAA-amino synthetase (*ClGH3.6*) (IAA deactivator) in the IAA signaling pathway and inhibiting its expression [10]. In maize, *ZmNAC128* and *ZmNAC130*, two NAC transcription factors that are specifically expressed in the maize endosperm, alter starch and protein accumulation in maize grains by binding to *cis*-acting elements (ACGCAA), thus regulating the expression of downstream genes [11]. More than 30 NAC transcription factors have been reported to be associated with leaf senescence in *A. thaliana*; for example, *ANAC092*, *ANAC029*, *ANAC059* and *ANAC016* are positive regulators of leaf senescence, while *ANAC042* and *ANAC083* negatively regulate leaf senescence [12].

Many studies have demonstrated that NAC transcription factors are not only widely involved in plant growth and development but can also respond to multiple abiotic stresses [7,13]. Drought and salt stress are major adverse factors affecting plant growth and development, and NAC transcription factors improve drought tolerance by activating downstream genes in response to drought stress [13]. In *A. thaliana*, *ANAC019/055/072* enhances drought resistance by mediating the ABA signaling pathway [14]. In *Oryza sativa* L., *OsNAC5*, *OsNAC6* and *OsNAC10* significantly enhance plant drought tolerance and increase yield under drought conditions [15,16,17]. Knockdown of *TaNAC071-a* in wheat was shown to obviously weaken drought tolerance in *Triticum aestivum* L., while overexpression of this gene was able to increase water use efficiency and activate the expression of stress response-related genes, resulting in significantly enhanced drought tolerance [18]. In *Solanum lycopersicum* L., *SlNAC35* enhances drought and salt tolerance in tomato by promoting root growth and development through an ABA-dependent pathway [19]. In *G. max, GmNAC06* enhances *G. max* salt tolerance by controlling the Na^+^/K^+^ ratio in roots and maintaining ion balance [20]. In *Malus domestica*, *MdNAC029* negatively regulates plant cold tolerance in a CBF-dependent manner by directly repressing *MdCBF1* and *MdCBF4* expression by binding to their promoters [21].

Alfalfa (*Medicago sativa* L.) is one of the most important legume forage grasses worldwide and is known as the king of forage grasses [22]. It is well known that cold stress, drought and salt stress severely restrict the normal growth and development of alfalfa and affect its final yield [23]. With the rapid development of sequencing technology and the accumulation of genomic as well as transcriptomic data, genome-wide identification and analysis of *NAC* transcription factor genes have been performed in many plants. However, since the alfalfa reference genome was not published until 2020 [24], the *NAC* gene family has not yet been characterized and analyzed at the genome-wide level in this plant.

In this study, a total of 421 *MsNAC* (NAC genes in the *M. sativa* genome) members were identified and characterized through basic bioinformatics analyses. This study analyzed the gene structure, motif composition, chromosome location and gene replication events of the *421 MsMACs*, and the evolutionary relationship between *M. sativa* and *A. thaliana*, *M. truncatula* (*Medicago truncatula* L.), and *G. max* (*Glycine max* L.) was examined. A quantitative real-time PCR (qRT–PCR) analysis was performed to examine the gene expression patterns in different tissues and their responses to cold, drought and salt stress. Through an overall expression analysis in alfalfa, the role of the *NAC* members in the different biological processes of alfalfa was determined. These results could provide valuable information for identifying candidate *MsNAC* genes involved in various abiotic stress responses in alfalfa.

## 2. Results

### 2.1. Identification of NAC Genes in the Alfalfa Genome

**Figure 1:**
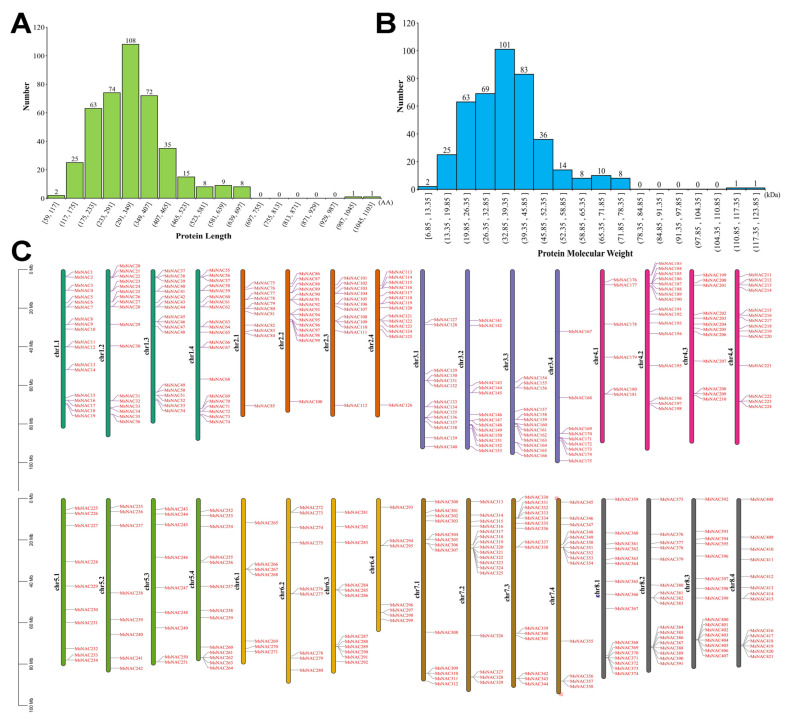
The characteristics and genome positions of MsNAC members in *M. sativa*. (**A**), Protein sequence length distribution; AA, amino acid. (**B**), The protein molecular weight distribution. (**C**), The position of *MsNAC* members in the alfalfa genome.

A total of 421 *NAC* genes were found in the alfalfa genome based on the HMM search and domain analysis. Basic information on these 421 members of the *NAC* gene family in alfalfa is listed in Table S2. As shown in Figure 1, the protein sequences ranged from 59 to 1090 in length, and nearly 25.7% of the members were between 291 and 349 in length (Figure 1A). The predicted protein molecular weights ranged from 6.85 kDa to 123.01 kDa and were mainly concentrated between 32.85 kDa and 39.35 kDa (101, 24%) (Figure 1B). Among these members, *MsNAC40* has the longest protein sequence length (1090 AA) and the largest protein molecular weight (123.01 kDa), while *MsNAC155* has the shortest protein sequence length (59 AA) and the smallest protein molecular weight (6.85 kDa).

The 421 members of the *NAC* gene family are distributed across the 32 chromosomes of the alfalfa reference genome (Figure 1C). The results revealed an average of 13 *NAC* genes on each chromosome. The chromosome with the highest number of *NAC* genes (20) was chr1.4, whereas the fewest, only 6, were found on chr4.1. The *NAC* gene distributions also presented clustering on some chromosomes, such as *MsNAC319–MsNAC325* on chr7.2 and *MsNAC369–MsNAC374* on chr8.1 (Figure 1C).

### 2.2. Motif Analysis of the Identified MsNAC Proteins

**Figure 2:**
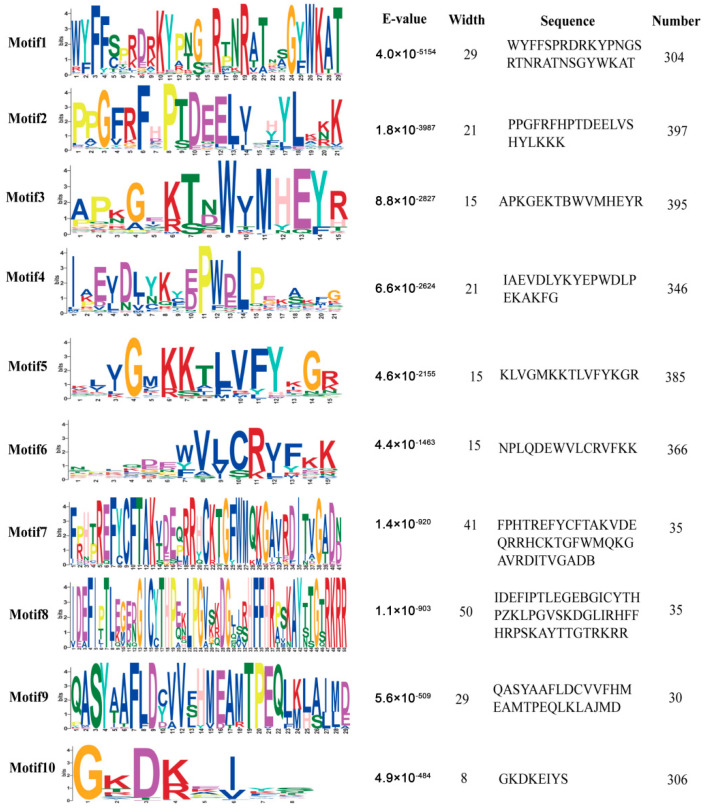
Conserved motif information for MsNAC proteins.

To identify the conserved motifs within the alfalfa NAC transcription factor gene family, MEME analysis was conducted. A total of 10 conserved motifs (named Motif1 to Motif10) were found among the MsNAC members; detailed information for each MsNAC member and motif are listed in Table S3 and Figure 2. The results showed that the sequence length of the 10 motifs varied from a minimum of 8 amino acids (Motif10) to a maximum of 50 amino acids (Motif8). Some motifs were conserved among most MsNAC members, while other motifs were unique to a few MsNAC members. For example, a total of 397 MsNAC members have Motif2, while only 30 MsNAC members have Motif9. Most MsNAC members contain Motif1, Motif2, Motif3, Motif4, Motif5, Motif6, and Motif10, while only 35, 35, and 30 MsNAC members contain Motif7, Motif8, and Motif9, respectively. Each member of the *MsNAC* gene family contain a minimum of 1 to a maximum of 7 of these motifs (Table S3); 57% of the MsNAC members contain seven different motifs, whereas MsNAC155 contains only one motif (Motif2). This variable motif distribution may create diversity in the function of the *MsNAC* gene family.

### 2.3. Phylogenetic Analysis

**Figure 3:**
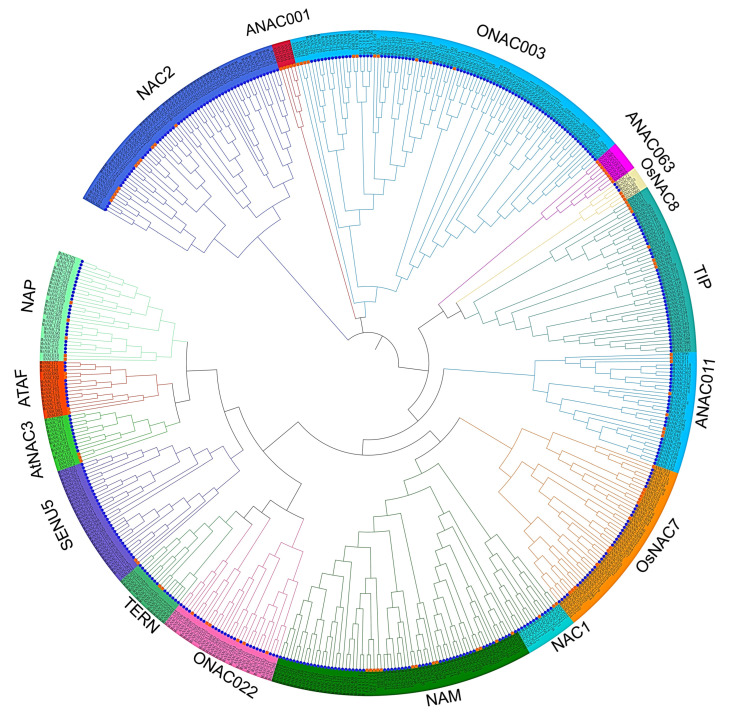
The phylogenetic tree constructed from 421 MsNAC proteins in *M. sativa* and 105 AtNAC proteins in *A. thaliana*. Different colors indicate different groups, and 16 groups were found.

To understand the structural classification of the *MsNAC* gene family, a neighbor-joining tree was constructed by combining the 421 MsNAC proteins and the 105 NAC proteins according to their homology to genes in *A. thaliana* with MEGA6.0 software [6]. The results showed that the 526 NAC proteins (421 from alfalfa and 105 from *A. thaliana*) were clustered into 16 subgroups according to the classification of AtNACs (Figure 3, Table S4). These results were consistent with those of previous studies in rice and *A. thaliana*. The 421 MsNAC proteins were distributed unequally among the 16 subgroups. There were 83 MsNAC members in Group 14, which was also named ONAC033, while no MsNAC members were found in Group 13 (named ANAC063) or Group 15 (named ANAC001). ANAC072, which is also named RD26, plays an important role in the plant response to drought stress and root development [14]. Here, MsNAC399, MsNAC381, MsNAC415, MsNAC367, MsNAC382, MsNAC414, MsNAC383, MsNAC91, MsNAC106, MsNAC80 and MsNAC119 were grouped with ANAC072. This result indicated that these MsNAC members may also respond to drought stress or other abiotic stresses in alfalfa.

### 2.4. Analysis of the Gene Duplication, Synteny and Evolution of the MsNACs

**Figure 4:**
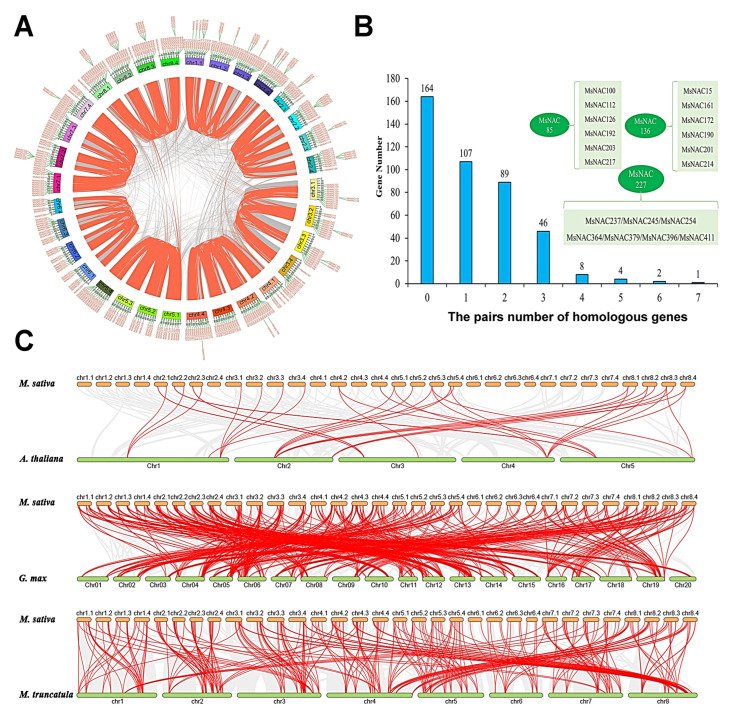
Duplication event analysis for the *MsNAC* gene family in the *M. sativa* genome and synteny analysis between alfalfa and the other three plant species. (**A**), The duplication events in the *M. sativa* genome; colored lines indicate *MsNAC* family members. (**B**), Statistical analysis of the duplication events; three duplication events (*MsNAC85*, *MsNAC136* and *MsNAC227*) are displayed. (**C**), Collinearity analysis between *M. sativa* and *A. thaliana*, *G. max* and *M. truncatula*. The colored lines indicate the NAC family members in different species.

As shown in Figure 4A and Table S5, a total of 494 pairs of homologous genes involving 257 *MsNAC* members were found in the alfalfa genome. Among these duplication events, 434 involved duplications of the four allelic chromosomes. For example, the paralogy of *MsNAC1* at 3.3 Mb on chr1.1 and *MsNAC20* at 3.8 Mb on chr1.2, as well as *MsNAC37* at 3.8 Mb on chr1.3 and *MsNAC55* at 3.4 Mb on chr1.4, arose from genome duplication events. Among the 421 *MsNAC* members, 164 genes were singletons in the alfalfa genome (Figure 4B). In addition, we found that some *MsNAC* genes had more than two paralogs in the alfalfa genome, such as *MsNAC85* and *MsNAC136,* which both have six paralogous genes, and *MsNAC227*, which has seven paralogs (Figure 4B).

To further understand the possible evolutionary events involving the *NAC* gene family in different crops, three comparative syntenic maps of *M. sativa* with *A. thaliana*, *G. max* and *M. truncatula* were constructed. The results showed 37 collinear *NAC* gene pairs between *M. sativa* and *A. thaliana*, 532 collinear *NAC* gene pairs between *M. sativa* and *G. max* and 306 orthologs between *M. sativa* and *M. truncatula* (Figure 4C, Table S6). The number of orthologous gene pairs between *MsNACs* and *AtNACs* was far fewer than the number between *MsNACs* and *G. max* *NAC* genes (*GmNACs*) or *MsNACs* and *M. truncatula* *NAC* genes (*MtNACs*), likely because *G. max*, *M. truncatula* and *M. sativa* are all legumes.

### 2.5. Expression Analysis of MsNAC Genes in Different Tissues

**Figure 5:**
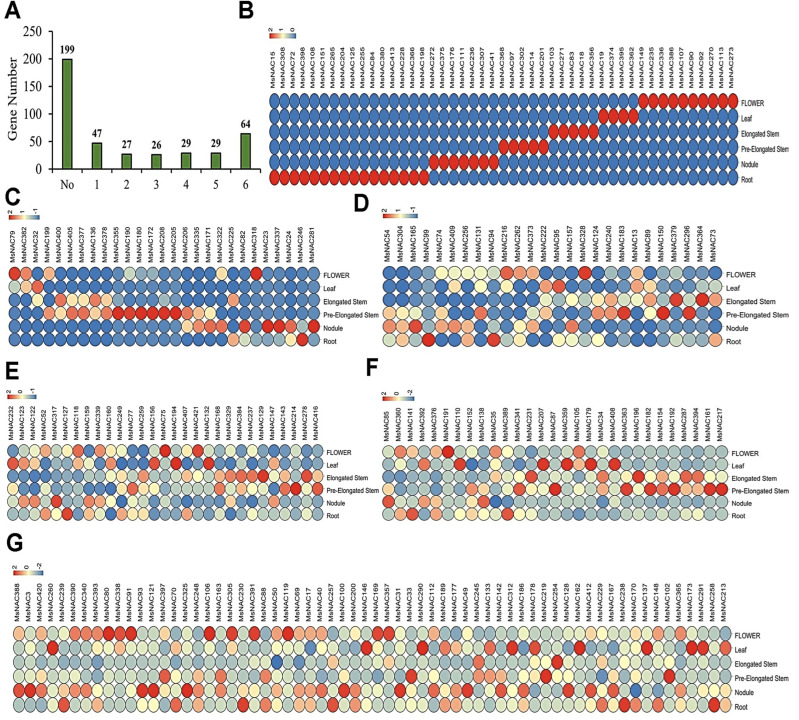
Expression analysis of *MsNAC* genes in different tissues (flower, leaf, elongated stem, pre-elongated stem, nodule and root). (**A**), Statistical analysis of the number of *MsNAC* genes expressed in different tissues. (**B**), The 47 *MsNAC* genes expressed in only one tissue. (**C**), The 27 *MsNAC* genes expressed in two tissues. (**D**), The 26 *MsNAC* genes expressed in three tissues. (**E**), The 29 *MsNAC* genes expressed in four tissues. (**F**), The 29 *MsNAC* genes expressed in five tissues. (**G**), The 64 *MsNAC* genes expressed in six tissues. The expression levels were normalized by row using the Z-Scores algorithm. The color scale at the top of the heatmap refers to the relative expression level, and the color gradient from blue to red presents an increasing expression level.

Whole-genome gene expression data are publicly available for several tissues in *M. sativa*, including flowers, leaves, elongated stems, pre-elongated stems, nodules and roots. As shown in Figure 5A, a total of 199 *MsNAC* genes were not expressed in the six tissues investigated in this study. These genes may be expressed in other tissues or specifically when alfalfa is under biological or abiotic stress. For the other 222 expressed *MsNAC* genes, the transcript abundances varied among different tissues, suggesting that the functions of these genes are obviously different. In this case, 47 *MsNAC* genes were expressed in only one tissue, showing obvious tissue expression specificity (Figure 5B). Among these 47 *MsNAC* genes, 10 (*MsNAC149*, *MsNAC235*, *MsNAC336*, *MsNAC386*, *MsNAC107*, *MsNAC90*, *MsNAC92*, *MsNAC270*, *MsNAC113*, and *MsNAC273*) were expressed only in flowers, and 4 (*MsNAC19*, *MsNAC374*, *MsNAC395*, and *MsNAC362*) were expressed only in leaves; 5, 5, 7 and 16 *MsNAC* genes were expressed exclusively in elongated stems, pre-elongated stems, nodules and roots, respectively (Figure 5B). There were 27 *MsNAC* genes expressed in two different tissues (Figure 5C). For example, *MsNAC79* and *MsNAC382* were expressed in both flowers and leaves. Moreover, 26, 29 and 29 different *MsNAC* genes were expressed in three, four and five different tissues, respectively (Figure 5D–F). In addition, we found that 64 *MsNAC* genes were expressed in all six tissues (Figure 5G). These results show that these *MsNAC* genes are broadly involved in the growth and development of alfalfa and play important roles.

### 2.6. Expression Analysis of MsNAC Genes under Cold Stress

**Figure 6:**
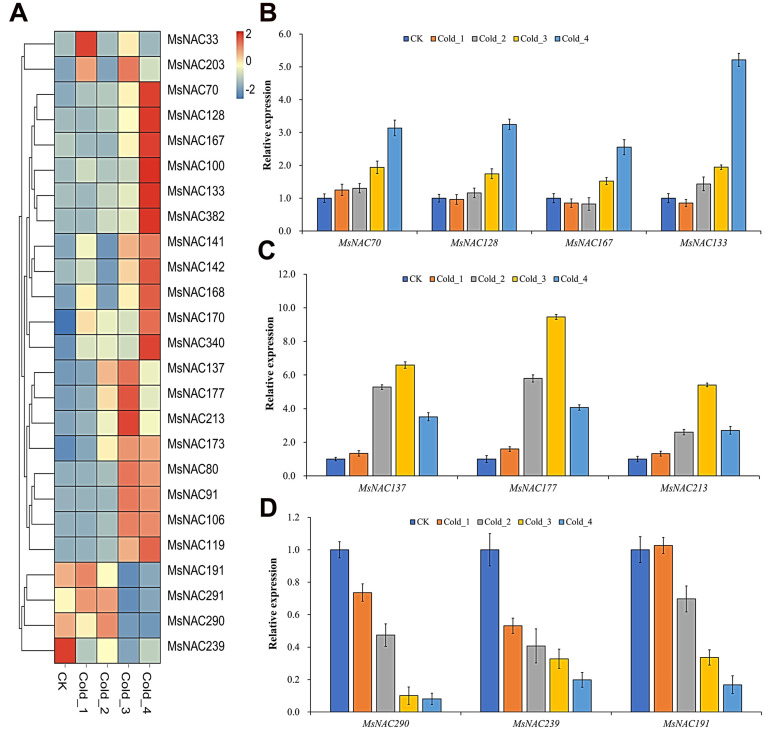
*MsNAC* genes that respond to cold stress. (**A**), Heatmap of the 25 *MsNAC* genes that respond to cold stress. The expression levels were normalized by row using the Z-Scores algorithm. The color scale at the right of the heatmap refers to the relative expression level, and the color gradient from blue to red presents an increasing expression level. (**B**), RT–PCR of *MsNAC70*, *MsNAC128*, *MsNAC167* and *MsNAC133*. (**C**), RT–PCR of *MsNAC290*, *MsNAC239* and *MsNAC191*. (**D**), RT–PCR of *MsNAC137*, *MsNAC177* and *MsNAC213*. CK was arbitrarily set to 1. Error bars represent the standard deviations of three technical replicates.

To study *MsNAC* gene expression in alfalfa under cold stress, we analyzed their transcript abundance changes in transcriptome and RT–PCR data. As shown in Figure 6A, the expression of 25 *MsNAC* genes changed significantly. Based on their expression patterns, these genes were clustered into two groups: the first group contained four genes (*MsNAC191*, *MsNAC291*, *MsNAC290* and *MsNAC239*) that were significantly downregulated under cold stress, and the second group contained 21 *MsNAC* genes that were upregulated under cold stress. The expression of the 21 *MsNAC* members in the second group peaked at different time points after cold stress. For example, the transcript abundance of *MsNAC33* peaked two hours after cold stress treatment (Cold_1), the transcript abundance of *MsNAC213* peaked 24 h after cold stress treatment (Cold_3), and the transcript abundance of *MsNAC70* increased with the extension of cold stress treatment time. To further validate this result, we performed RT–PCR. The results showed that the expression of *MsNAC70*, *MsNAC128*, *MsNAC167* and *MsNAC133* increased with the extension of cold stress treatment time (Figure 6B). The expression of *MsNAC290*, *MsNAC239* and *MsNAC191* decreased with prolonged cold stress, and the expression of *MsNAC137*, *MsNAC177* and *MsNAC213* initially rose and later fell after cold stress treatment (Figure 6C,D). These results were consistent with those of the transcriptome data analysis.

### 2.7. Expression Analysis of MsNAC Genes under Drought Stress

**Figure 7:**
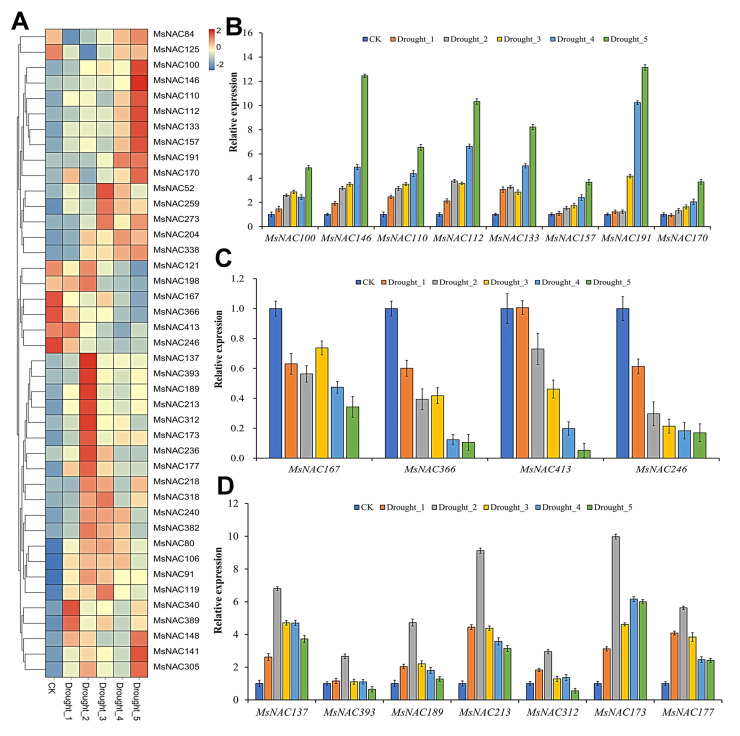
*MsNAC* genes that respond to drought stress. (**A**), Heatmap of the 42 *MsNAC* genes that respond to drought stress. The expression levels were normalized by row using the Z-Scores algorithm. The color scale at the right of the heatmap refers to the relative expression level, and the color gradient from blue to red presents an increasing expression level. (**B**), RT–PCR of *MsNAC100*, *MsNAC146*, *MsNAC110*, *MsNAC112*, *MsNAC133*, *MsNAC157*, *MsNAC191* and *MsNAC170*. (**C**), RT–PCR of *MsNAC167*, *MsNAC366*, *MsNAC413* and *MsNAC246*. (**D**), RT–PCR of *MsNAC137*, *MsNAC393*, *MsNAC189*, *MsNAC213*, *MsNAC312*, *MsNAC173* and *MsNAC177*. CK was arbitrarily set to 1. Error bars represent the standard deviations of three technical replicates.

As shown in Figure 7A, a total of 42 *MsNAC* genes responded to drought stress. Based on their expression patterns under drought stress, the 42 *MsNAC* members were clustered into different groups. Among these genes, the transcript abundance of several genes continued to increase under drought stress (*MsNAC100*, *MsNAC146*, *MsNAC110*, *MsNAC112*, *MsNAC133*, *MsNAC157*, *MsNAC191*, and *MsNAC170*), while others continued to decrease in abundance (*MsNAC100*, *MsNAC146*, *MsNAC110*, *MsNAC121*, *MsNAC198*, *MsNAC167*, *MsNAC366*, *MsNAC413* and *MsNAC246*); additionally, some members first increased and then decreased in abundance (*MsNAC137*, *MsNAC393*, *MsNAC189*, *MsNAC213*, *MsNAC312*, *MsNAC173*, *MsNAC236* and *MsNAC177*). The RT–PCR results were consistent with the transcriptome analysis results (Figure 7B–D).

### 2.8. Expression Analysis of MsNAC Genes under Salt Stress

**Figure 8:**
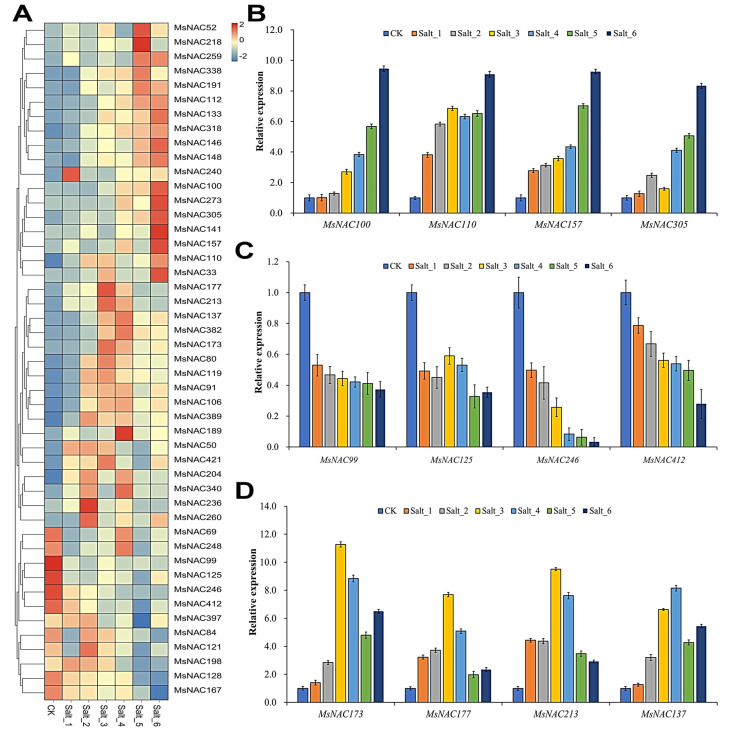
*MsNAC* genes that respond to salt stress. (**A**), Heatmap of the 47 *MsNAC* genes that respond to salt stress. The expression levels were normalized by row using the Z-Scores algorithm. The color scale at the right of the heatmap refers to the relative expression level, and the color gradient from blue to red presents an increasing expression level. (**B**), RT–PCR analysis of *MsNAC100*, *MsNAC110*, *MsNAC157* and *MsNAC305*. (**C**), RT–PCR of *MsNAC99*, *MsNAC125*, *MsNAC246* and *MsNAC412*. (**D**), RT–PCR of *MsNAC137*, *MsNAC177*, *MsNAC213*, *MsNAC312* and *MsNAC137*. CK was arbitrarily set to 1. Error bars represent the standard deviations of three technical replicates.

As shown in Figure 8A, 47 *MsNAC* members responded to salt stress. Among the 47 *MsNAC* genes, 12 (*MsNAC69*, *MsNAC248*, *MsNAC99*, *MsNAC125*, *MsNAC246*, *MsNAC412*, *MsNAC397*, *MsNAC84*, *MsNAC121*, *MsNAC198*, *MsNAC128* and *MsNAC167*) were downregulated under salt stress, and the other 35 were upregulated under salt stress. The results of RT–PCR showed that *MsNAC100*, *MsNAC110*, *MsNAC157* and *MsNAC305* were upregulated under salt stress, *MsNAC99*, *MsNAC125*, *MsNAC246* and *MsNAC412* were downregulated under salt stress, and *MsNAC173*, *MsNAC177*, *MsNAC213* and *MsNAC137* showed an initial elevation and subsequent decrease under salt stress (Figure 8B–D).

### 2.9. MsNAC Genes Are Involved in Various Abiotic Stresses

**Figure 9:**
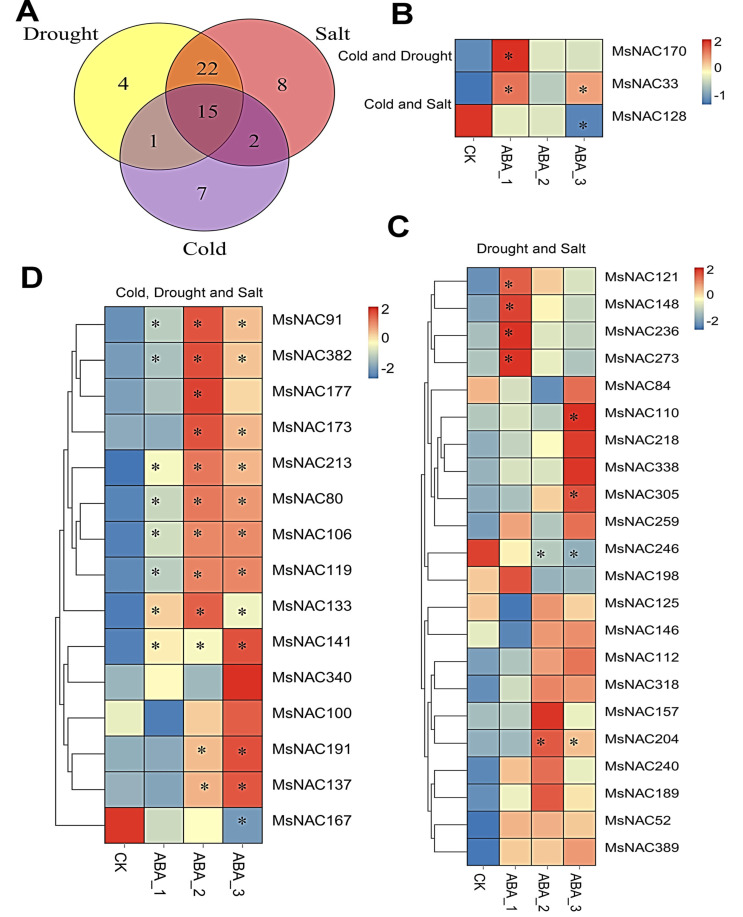
*MsNAC* genes that respond to multiple stresses. (**A**), Venn diagram of *MsNAC* genes responding to cold, drought and salt stress. (**B**), Three *MsNAC* genes (*MsNAC170,* which responded to cold and drought stress, and *MsNAC33* and *MsNAC128,* which responded to cold and salt stress) also responded to ABA treatment. (**C**), Expression analysis of 22 drought- and salt-responsive *MsNAC* genes under ABA treatment. (**D**), Expression analysis of 15 cold-, drought- and salt-responsive *MsNAC* genes under ABA treatment. The expression levels were normalized by row using the Z-Scores algorithm. The color scale at the right of the heatmap refers to the relative expression level, and the color gradient from blue to red presents an increasing expression level. * indicates a significant difference in expression based on padj < 0.05 and |log_2_FC| ≥ 1.

To understand which *MsNAC* members are involved in the responses to two or more abiotic stresses, we further analyzed the *MsNAC* genes that were differentially expressed under cold, drought, and salt stresses. As shown in Figure 9A, two *MsNAC* genes (*MsNAC33* and *MsNAC128*) responded to both cold and salt stress, and *MsNAC170* responded to both cold and drought stress. A total of 22 *MsNAC* genes responded to both drought and salt stress. Finally, 15 *MsNAC* genes responded to all three stresses.

Previous studies have shown that NAC transcription factors mediate the plant response to abiotic stress through both ABA and non-ABA pathways [7,12]. To elucidate whether similar molecular mechanisms exist for the *MsNAC* genes in alfalfa, we analyzed the changes in the transcript abundance of these genes after ABA treatment. The results showed that *MsNAC170,* which responded to cold and drought stress, and *MsNAC33* and *MsNAC128,* which responded to cold and salt stress, also responded to ABA treatment (Figure 9B). Of the 22 *MsNAC* genes that responded to drought and salt stress, 8 *MsNAC* genes (*MsNAC121*, *MsNAC148*, *MsNAC236*, *MsNAC273*, *MsNAC110*, *MsNAC305*, *MsNAC246* and *MsNAC204*) responded to ABA treatment (Figure 9C). Of the 15 *MsNAC* genes that responded to cold, drought and salt stress, 13 also responded to ABA treatment (Figure 9D). These results indicated that these *MsNAC* genes may also mediate abiotic stress responses through both ABA and non-ABA pathways, similar to *NACs* in other plants.

## 3. Discussion

The NAC transcription factor family, which has been proven by several studies to be widely involved in plant growth and development in response to abiotic stresses, is one of the important gene families for stress resistance improvement in crops [25]. To date, the identification of *NAC* gene family members at a genome-wide level has been accomplished in many plants [6,26,27,28,29,30,31]. For example, 105 *NAC* genes were identified in *A. thaliana*, 151 *NAC* genes in rice, 173 *NAC* genes in *G. max*, and 148 *NAC* genes in maize [6,26,27]. However, a systematic analysis and in-depth study of the *NAC* gene family at the whole-genome level is still lacking in alfalfa. In this study, we identified a total of 421 *NAC* gene members in the alfalfa reference genome (Figure 1 and Table S1). Basic bioinformatic analysis, sequence analysis, evolution analysis and expression analysis were conducted in this study. The results indicated that the *MsNAC* genes play important roles in the normal growth and development of alfalfa and may be important for improving alfalfa resistance to various abiotic stresses, such as cold, drought and salt.

Based on the functional characterization of homologs in different species, phylogenetic tree analysis was used to help predict gene function. Many studies have shown that NAC transcription factors regulate root growth in Arabidopsis and rice [32,33,34]. For example, *ANAC032* regulates root growth in Arabidopsis by upregulating MYB30 and other target genes [35]. Here, we found that MsNAC177, MsNAC213, MsNAC189, MsNAC137, MsNAC151, MsNAC162, MsNAC173, MsNAC134, MsNAC157, MsNAC146 and MsNAC133 clustered into the same group (named ATAF) as ANAC032 (Figure 3). Among these *MsNAC* genes, only *MsNAC151* was specifically expressed in alfalfa roots (Figure 5). Thus, based on phylogenetic analysis and expression analysis, we inferred that *MsNAC151* may also play an important role in alfalfa root development.

Many NAC transcription factors are involved in responses to various abiotic stresses, such as cold, drought and salt [7,12]. The overexpression of three genes, *ANAC019*, *ANAC055* and *ANAC072*, significantly improved the drought resistance of the plants and regulated the expression of ERD1 and its downstream genes by binding to the CATGTG core region of the promoter of the *ERD1* gene, which is involved in the drought stress response [14]. In the present study, 25, 42 and 47 *MsNAC* genes were found to respond to cold, drought and salt stress, respectively (Figure 6, Figure 7 and Figure 8). The phylogenetic analysis showed that 11 MsNAC members (MsNAC399, MsNAC381, MsNAC415, MsNAC367, MsNAC382, MsNAC414, MsNAC383, MsNAC91, MsNAC106, MsNAC80 and MsNAC119) were clustered into the AtNAC3 group with ANAC019, ANAC055 and ANAC072 (Figure 3). The expression of *MsNAC382*, *MsNAC106*, *MsNAC91*, *MsNAC80* and *MsNAC119* was significantly upregulated under drought stress (Figure 7). Therefore, these genes may be important candidate genes for the drought stress response in alfalfa. Some NAC transcription factors are involved in the responses to two or more abiotic stresses in other plants. For example, overexpression of potato *StNAC053* could improve salt and drought tolerance, and overexpression of *SNAC1* in rice enhanced drought and salt tolerance in field tests by improving root development [36,37]. This phenomenon was also found in the *MsNAC* gene family. A total of 22 *MsNAC* genes responded to both salt and drought stress, and 15 *MsNAC* genes responded to cold, salt and drought stress (Figure 9).

Alfalfa is one of the most important forage crops in the world [22]. It is known as the ‘king of forage grasses’ and is considered a high-quality forage for herbivores such as cows due to its high fitness, stress resistance, high yield, abundant nutrient content and high palatability [24]. Similar to other crops, alfalfa is often subjected to various abiotic stresses, such as cold, drought or salt stress, during growth and development, causing yield and quality losses [23]. Therefore, mining alfalfa stress tolerance genes and developing molecular breeding strategies are important for the genetic improvement of alfalfa. The NAC transcription factor family, as one of the important gene families in plants, plays important roles in plant growth and responses to different abiotic stresses. The results of this study provide useful information about the *MsNAC* gene family, and the functions of the *MsNACs* identified to respond to various stresses should be confirmed by more experiments in the future.

## 4. Methods and Materials

### 4.1. Plant Materials and Growth Conditions

The alfalfa seeds (Cultivar Zhongmu No. 1) were preserved in our laboratory at the Institute of Animal Science of the Chinese Academy of Agricultural Sciences. The seeds were treated at 4 °C for 3 days before germination. Then, the seedlings were placed in a greenhouse at 24 °C (day)/20 °C (night) with a 16 h light/8 h dark photoperiod in the hydroponic culture medium for two weeks. For cold stress, the leaves were placed at 4 °C and collected at five time points (0 h as CK, 2 h as Cold_1, 6 h as Cold_2, 24 h as Cold_3 and 48 h as Cold_4). In this study, 400 mM mannitol was used to simulate drought stress, and the root tips were collected at six time points after mannitol treatment (0 h as CK, 1 h as Drought_1, 3 h as Drought_2, 6 h as Drought_3, 12 h as Drought_4 and 24 h as Drought_5). NaCl (250 mM) was used to simulate salt stress, and the root tips were collected at seven time points after NaCl treatment (0 h as CK, 0.5 h as Salt_1, 1 h as Salt_2, 3 h as Salt_3, 6 h as Salt_4, 12 h as Salt_5 and 24 h as Salt_6). Three replicates with five single seedlings in each replicate were collected for each condition. The samples were stored at −80 °C for further RT–PCR experiments.

### 4.2. Identification of MsNAC Gene Family Members in the Alfalfa Reference Genome

We obtained the alfalfa reference genome sequence (Xinjiangdaye) from Figshare (https://figshare.com, accessed on 14 November 2021) [24]. The hidden Markov model (HMM) profile for the NAC domain (PF02365) was obtained from the Pfam database (https://pfam.xfam.org/, accessed on 10 April 2022). Then, we used HMMER software with a cutoff E-value > e^−10^ to identify alfalfa protein sequences that matched the NAC HMM profile. These candidate MsNAC protein sequences were further analyzed by using the conserved domain-search online program (https://www.ncbi.nlm.nih.gov/, accessed on 10 April 2022) to identify the NAC domain.

### 4.3. Basic Bioinformatics Analysis of MsNACs

The cDNA sequence length, genome position, and protein length of the identified *MsNACs* were obtained from the Xinjiangdaye reference genome gff file by using TBtools software [38]. The theoretical isoelectric points (pIs) and protein molecular weights (MWs) were analyzed with ExPASy online (https://web.expasy.org/compute_pi/, accessed on 29 April 2022). The determination of the conserved motifs in the MsMAC proteins was conducted by the MEME online program (http:/meme.nbcr.net/meme/intro.html, accessed on 30 April 2022), and the parameters were set to an optimum mode width of 6 to 200 and a maximum number of motifs of 10.

### 4.4. Phylogenetic Analysis, Gene Duplication and Synteny Analysis

The NAC protein sequences for the phylogenetic tree were obtained from the UniProt database (https://www.UniProt.org, accessed on 2 May 2022). The multiple amino acid sequences of identified *MsNAC* genes were aligned using Clustalx2.0 software with the default parameters. Phylogenetic trees comparing *M. sativa* and *A. thaliana* were constructed with the NJ method, and the specific parameters were the Poisson model and 1000 bootstrap replications in MEGA software. The NAC protein sequences from *M. sativa* and *A. thaliana* were also aligned using the Clustalx2.0 program before the phylogenetic tree was constructed. According to the classification of AtNAC, all the identified *MsNAC* genes were divided into 16 groups [5]. Information concerning the chromosomal location of *MsNAC* genes, including the chromosome length, gene direction, and gene start and stop positions, was obtained from the alfalfa genome database. MCScanX software was used to analyze the *MsNAC* replication events and detect collinear regions between *MsNACs* and collinear blocks of *NAC* genes within *A. thaliana*, *M. truncatula*, and *G. max* [39]. All function and chromosomal location information was obtained by TBtools software [38].

### 4.5. Transcriptome Data Collection and Analysis

Transcriptomic data for six alfalfa tissues (flower, leaf, elongated stem, pre-elongated stem, nodule and root) were collected from the NCBI database (SRP055547) [40]. Transcriptomic data for alfalfa *MsNAC* genes exposed to cold, drought, salt and ABA treatments were collected from the NCBI database (SRR7091780–SRR7091794 and SRR7160313–SRR7160357) [41]. TopHat2 was used to map the obtained clean reads to the reference genome (Xinjiangdaye) [42]. The FPKM value was used to estimate the gene expression level, and the differentially expressed genes were obtained by DESeq with padj < 0.05 and |log_2_FC| ≥ 1 [43]. TBtools software was used for data visualization [38].

### 4.6. RT–PCR Analysis

Total RNA was extracted from the leaf and root tip samples in this study by using TRIzol reagent according to the manufacturer’s instructions. Then, the corresponding cDNA was obtained by using the EasyScript First-Strand cDNA Synthesis kit. The RT–PCR primers for these *MsNAC* genes were designed by using Primer 5.0 software [44]. The RT–PCR experiment was conducted by using SYBR Premix Ex Taq (Takara, Japan) on the 7500 Real-Time PCR system (Applied Biosystems, CA, USA). Three replicates were designed for each sample, and alfalfa actin gene expression was used for data normalization. The 2^−ΔΔCt^ method was used to calculate the relative gene expression levels of the *MsNAC* genes [45]. All the primers used in this study are listed in Table S1.

## 5. Conclusions

In this study, a total of 421 *NAC* genes (*MsNAC*s) were identified from the alfalfa “Xinjiangdaye” reference genome. The protein sequences ranged from 59 to 1090 aa in length. The predicted protein molecular weights ranged from 6.85 kDa to 123.01 kDa. The 421 members of the *NAC* gene family are distributed across the 32 chromosomes of the alfalfa reference genome. Expression analysis showed that 47 *MsNAC* genes had tissue-specific expression, and 64 *MsNAC* genes were expressed in all tissues. The transcriptomic profiles of the genes were very different, indicating that these *MsNAC* genes have various functions in alfalfa growth and development. We identified 25, 42 and 47 *MsNAC* genes that respond to cold, drought and salt stress based on transcriptome data analysis and real-time quantitative PCR (RT–qPCR). Furthermore, 22 *MsNAC* genes were found to respond to both salt and drought stress, and 15 *MsNAC* genes were found to respond to cold, salt and drought stress. The results of this study could provide valuable information for further functional analysis of *MsNAC* genes and for the improvement of stress resistance in alfalfa.

## Data Availability

Not applicable.

## Supplementary Materials

The following supporting information can be downloaded at: https://www.mdpi.com/article/10.3390/ijms231710015/s1.
